# “It’s not just about walking.....it’s the practice nurse that makes it work”: a qualitative exploration of the views of practice nurses delivering complex physical activity interventions in primary care

**DOI:** 10.1186/s12889-015-2568-6

**Published:** 2015-12-12

**Authors:** Carole Beighton, Christina Victor, Rebecca Normansell, Derek Cook, Sally Kerry, Steve Iliffe, Michael Ussher, Peter Whincup, Julia Fox-Rushby, Alison Woodcock, Tess Harris

**Affiliations:** Faculty of Health, Social Care & Education, Kingston & St George’s University of London, London, SW17 ORE UK; College of Health and Life Sciences, Brunel University London, Middlesex, UB8 3PH UK; Population Health Research Institute, St Georges University of London, London, SW17 ORE UK; Pragmatic Clinical Trials Unit, Queen Mary’s University of London, London, E1 2AT UK; Research Department of Primary Care & Population Health, University College, London, NW3 2PF UK; Health Economics Research Group, Brunel University, Uxbridge, UB8 3PH UK; Independent Psychology Research Consultant, Southampton, UK

**Keywords:** Practice nurse, Primary care, Randomised controlled trial, Walking intervention, Physical activity, Behaviour change techniques

## Abstract

**Background:**

Physical activity (PA) is important for physical and mental health in adults and older adults. Interventions incorporating theory-based behaviour change techniques (BCTs) can be useful in helping people to increase their PA levels and can be delivered by practice nurses in primary care. We undertook two primary care based complex walking interventions among adults and older adults. Both interventions were underpinned by BCTs and delivered by practice nurses and we sought their views and experiences of delivering over 1400 complex PA consultations.

**Methods:**

Semi structured interviews with two practice nurse groups (*n* = 4 and *n* = 5) and two individual interviews (total *n* = 11) were conducted by independent facilitators; audio-recorded, transcribed verbatim and analysed using thematic analysis.

**Results:**

Five key themes emerged as enablers and/or barriers to delivering the intervention: preparation and training; initial and ongoing support; adherence to the protocol; the use of materials and equipment; and engagement of participants. The themes were organised into a framework of ‘pre-trial’ and ‘delivery of the intervention’. Two additional ‘post-trial’ themes were identified; changed practice and the future feasibility of the intervention. Nurses believed that taking part in the trial, especially the BCT training, enhanced the quality and delivery of advice and support they provided within routine consultations, although the lack of time available routinely makes this challenging.

**Conclusion:**

Delivering an effective behaviour change intervention in primary care requires adequate training and support for practice nurses both initially and throughout the trial as well as adequate consultation time. Enhanced skills from participating in such trials can lead to long-term changes, including more patient-centred consulting.

**Trial registration:**

PACE-Lift ISRCTN 42122561, PACE-UP ISRCTN 98538934.

## Background

Worldwide, physical inactivity is the fourth leading cause of death [1] and directly contributes to one in six deaths in the UK [2]. Approximately a quarter of adults are classified as ‘inactive’ as they do not achieve 30 min of PA a week [3] and the proportion of older adults achieving recommended levels is much lower [4]. Increasing PA has the potential to significantly improve life expectancy, reduce all-cause mortality and improve both physical and mental wellbeing [4].

It is recommended that inactive adults and older adults are identified and assessed either opportunistically or during routine general practice (GP) consultations within primary care [3, 5] as the majority of National health service (NHS) patient contacts take place in this setting [6]. Within the United Kingdom, the practice nurse plays an central role in patient care within GP surgeries and health centres including assessment and treatment of minor ailments, undertaking investigatory procedures, vaccinations, lifestyle education, health promotion and chronic disease prevention and management; a role broadly comparable to general practice nurses in Australia, family practice registered nurses in Canada and family nurse practitioners in the USA.

All practice nurses routinely identify, engage and support patients with health behaviour change interventions informed by research findings as part of primary or secondary prevention strategies [7, 8], by ‘making every contact count’ [9]. This might include NHS health checks [10], diabetes care and smoking cessation advice. Although not an integral part of their role to take part in clinical research studies which includes randomized controlled trials, some practice nurses do successfully participate in research, including delivering complex behaviour change interventions [11–14].

## Background to study

Between 2011 and 2014, ten general practices and 12 practice nurses from within Oxfordshire and Berkshire West Primary Care Trusts and across South West London participated in two large randomised controlled trials of pedometer-based walking interventions, the PACE-Lift and PACE-UP trials. Both trials investigated whether adults could increase their PA levels using complex interventions to increase walking, provided on an individual basis within practice nurse physical activity consultations and practice nurses, with their experience and expertise in supporting behaviour change, were identified as the most appropriate health care professionals to deliver the interventions.

The interventions in both trials were based on pedometer step-count feedback (and accelerometer feedback in the PACE-Lift trial) combined with practice nurse PA consultations. The interventions incorporated BCTs such as goal-setting, self-monitoring, building self-efficacy and relapse prevention. The complex interventions were well accepted by participants with 86% (129/150) attending all four nurse consultations in PACE-Lift and 74% (255/346) attending all three sessions in PACE-UP with the practice nurses delivering approximately 1400 PA consultations across the two trials. Both trials were successful and showed that the interventions were effective in increasing both average daily step-counts and time in moderate-to-vigorous physical activity at both 3 and 12 months, in 60–75 year olds in PACE-Lift [15] and 45–75 year olds in PACE-UP [16]. The trial protocols provide full details of each trial, the BCTs undertaken and procedures to ensure trial fidelity [17, 18]. A summary of both trials is provided in Table 1.

**Table 1:** Summary of the PACE-Lift and PACE-UP trials

Trial	PACE-Lift	PACE- UP
	Recruitment: October 2011–October 2012	Recruitment: October 2012–October 2013
	12 month follow-up: October 2012-October 2013	12 month follow-up: October 2013-October 2014
Study Design	2-arm parallel design, cluster randomised controlled trial with intervention and control (usual care) arms plus process and qualitative evaluations. Randomised by household^a^	3-arm randomised controlled trial with 12 month follow-up and health economic and qualitative evaluations. Randomised by household^a^
Aims	To determine if an intervention based on pedometer and accelerometer feedback combined with practice nurse PA consultations in primary care is effective in helping people aged 60–74 years to increase their PA levels over a 3 month period and to maintain any increase over a year	To determine whether inactive patients aged 45–74 years can increase their PA by being given a pedometer with a diary and written guidelines and whether additional individual, tailored, support from a practice nurse increases any benefits over a 3 month period. Main outcome assessed at 12 months.
Practices:	3 GP practices in Oxfordshire and Berkshire, UK	7 GP Practices in South West London, UK
Practice nurses	4	8
Participant eligibility	Able to walk outside and had no contra-indications to increasing PA.	Able to walk outside and had no contraindications to increasing PA and reporting not achieving the current UK PA guidelines
Age range	60–75 years	45–75 years
Participants	Randomly selected	Randomly selected
	*n* = 298 (138 m, 160 f)	*n* = 1023 (367 m, 656 f)
	Couples 99/298 (33 %) (50 couples, 1 person withdrew)	Couples 209/1023 (20 %) (105 couples, 1 person withdrew)
	Randomised to:	Randomised to:
	• Pedometer + accelerometer intervention + nurse support (*n* = 150) • control (*n* = 148)	• pedometer intervention + nurse support (*n* = 346) • postal pedometer intervention (*n* = 339) • control (*n* = 338)
Intervention	The intervention group received four practice nurse PA consultations over a 12 week period (weeks 1, 3, 7 and 11). These incorporated behaviour change techniques, feedback on pedometer step-counts and accelerometer PA intensity, a PA diary and individually tailored PA plan.	The Intervention group (pedometer plus nurse support) received a pedometer and diary and three individually tailored PA practice nurse consultations (weeks 1, 5, 9). They were supported to follow a 12-week pedometer-based walking programme, using strategies such as self-monitoring, goal-setting, boosting motivation and anticipation of set-backs.
	The accelerometer required downloading in the consultation and graphs of PA intensity were shown to participants.	The pedometer Intervention group were posted out a pedometer, a diary, and written instructions for a 12-week pedometer-based walking programme, based on their own baseline blinded pedometer step-count. Followed-up at 3, 6, 9 and 12 months. There were no meetings with the practice nurse.
	The control group continued usual PA	The control group continued usual PA
	In both trials, participants were provided with an individualised PA plan by the practice nurses, starting from where the individual was and increasing both step count and time spent in MVPA	
Nurse time commitment	Each consultation was approx 30 min. Including other administrative duties total time approximately 2 h a week per practice	Each consultation was 20–30 min. Including other administrative duties total time approximately 2 h a week per practice
Outcome assessment	Main outcome assessment (7 day accelerometry to give average daily step-count and average weekly time in moderate to vigorous physical activity) at 3 months (face to face) then postal assessment at 12 months.	Main outcome assessment (7 day accelerometry to give average daily step-count and average weekly time in moderate to vigorous physical activity) at 12 months (face to face) interim postal assessment at 3 months.
Nurse Consultations	129/150 (86 %) attended all 4 nurse consultations	255/346 (74 %) attended all 3 nurse consultations
Full trial protocol:	Harris et al. (2013a) [17]	Harris et al. (2013b) [18]
	http://www.biomedcentral.com/1471-2458/13/5	http://www.trialsjournal.com/content/14/1/418

^a^Couples who were recruited were offered the choice to be seen together (double appointment) or separately

**Fig. 1:**
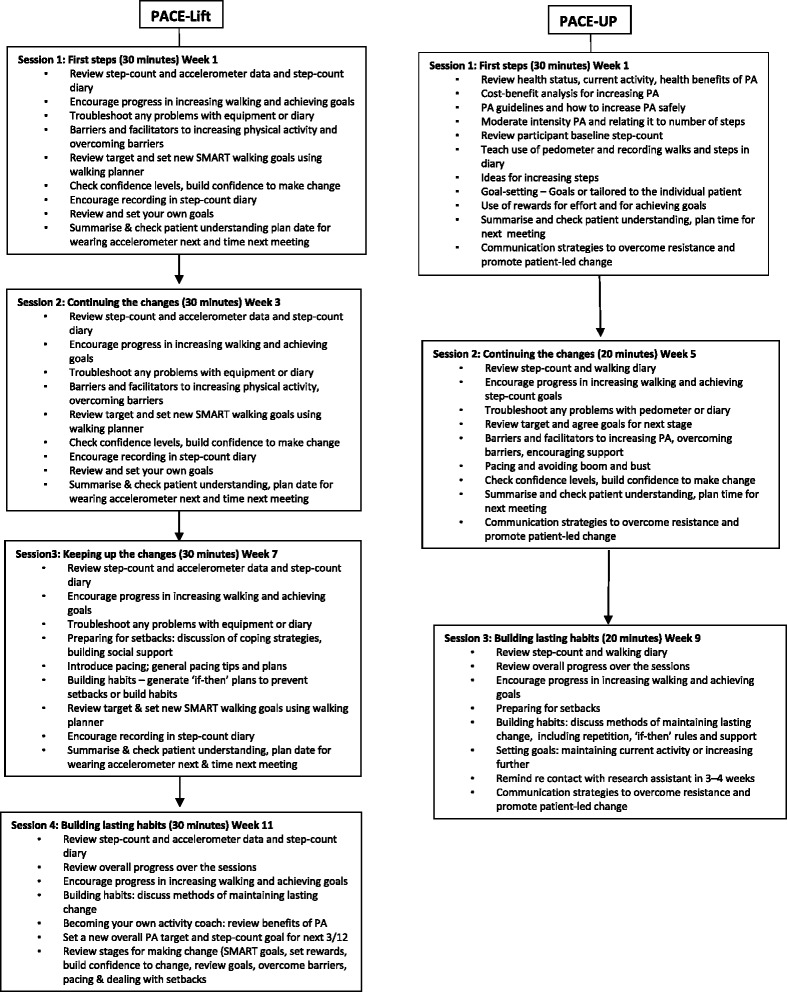
Details of the PACE-Lift and PACE-UP practice nurse physical activity consultation schedules

Initial training and continuing support for the practice nurses were considered essential to the success of our trials and preparation covered the theoretical framework, PA guidelines, benefits and safe ways of increasing PA, data recording, adverse event reporting, using the pedometers, accelerometers, diaries and also unique challenges, such as working with couples within a single consultation. Training in the specific BCTs was delivered by two national trainers with practice nurse training experience [19]. The techniques were based on evidence from a range of psychological theories [20, 21] and methods intended for NHS behaviour change programmes [22, 23] with adaptation for use in these trials to focus on PA and the use of pedometers. A summary of the nurse consultation guides for both trials is shown in Fig. 1.

Training for both trials followed a similar pattern. There was an initial meeting individually with nurses in their practices to introduce the trial and equipment etc.; a full day training session with the BCT trainers, chief investigator, trial manager and research assistants before seeing any patients. Three further half day sessions were arranged with the same personnel spread over 12 months to get feedback from nurses on the trial and provide further support on BCTs, communication skills and trouble shoot any problems. As well as the formal training events, nurses were in regular email contact with research assistants and a sample of their consultations were audio-recorded to allow individual feedback from the BCT trainer.

Qualitative explorations from the perspectives of those delivering and receiving the intervention are recommended to provide a more in-depth understanding of complex interventions [24, 25], enabling the intervention to be reproduced or adapted for the purposes of further research or for larger scale implementation [26, 27]. To date we have reported the main results of both PACE-Lift and PACE-UP trials [15, 16], the reasons for non-participation in both trials [28, 29] and the views of intervention participants from the PACE-UP trial [30]. The aim of this paper is to provide an additional layer of evaluation by exploring the views of the practice nurses, focusing upon the perceived enablers and barriers to delivering the complex PA interventions, identifying the benefits they gained as practitioners from participating in the trial and their evaluation of the acceptability of the intervention for use within routine PA consultations in a GP setting.

## Methods

We took a pragmatic approach, inviting the 12 trial practice nurses to attend group interviews. Qualitative interviews to gain the perspective of the people who implemented the intervention are recommended as the best approach for identifying barriers and facilitators in the delivery of a complex intervention [31]. Eleven nurses participated: all four PACE-Lift nurses attended the first group interview and five PACE-UP nurses attended the second. Two PACE-UP nurses were interviewed individually as they were unable to attend and one PACE-UP nurse was not available for interview. A semi-structured interview guide was developed by the research team (Appendix 1). The nurses gave verbal consent to participate and to be audio-recorded and confidentiality and anonymity were guaranteed (names were disassociated and aliases used for individuals and places). The two group interviews were led by experienced facilitators (CV, AW assisted by CB and RN) and the two individual PACE-UP nurse interviews were conducted by RN who had also participated in the PACE-UP focus group to ensure consistency in questioning.

Ethical approval to conduct the nurse interviews was gained within the main ethical approval for both PACE studies. PACE-Lift; Oxfordshire Research Ethics Committee C (11/H0606/2), PACE-UP; London Research Ethics Committee (Hampstead) (12/LO/0219).

### Analyses

The audio-files were transcribed verbatim and checked for accuracy. Checking of transcripts and coding the transcript themes was guided by thematic analysis [32] undertaken independently by a minimum of three researchers given the complexity of group interview analysis [33]. Areas of disagreement were discussed within the wider research team to ensure a consensus. To assure the reliability of the coding system; rater reliability, rather than inter-rater reliability, was considered more important within this process as the main focus was to elicit themes across the group discussion rather than individual member opinions. Constant referral back to the audio recordings, re-reading of the transcripts and field notes was made to ensure that the ‘group impact’ was accounted for [34].

The individual interviews were analysed using the same methods. The researchers were mindful when undertaking the analyses that group interviews reflect a generalised understanding whilst individual interviews provide more personal views and experiences. However, similar interpretations and themes emerged from both types of interview and referral to field-notes throughout the process enhanced the trustworthiness of the findings through data triangulation, with both providing the “same story, same meaning” [35, 36].

## Results

**Fig. 2:**
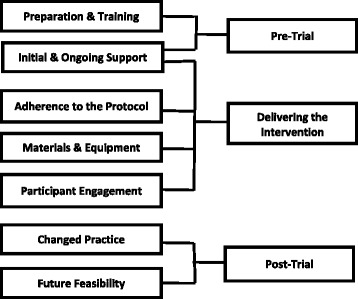
Key themes identified related to the trial phases

All 11 participants were registered nurses, had been qualified for between 3 and 33 years and had worked in general practice as band 5–7 practice nurses for between 3 and 24 years. Six had been involved in a previous randomised controlled trial and five had already undertaken some form of BCT training. The mean length of the two group interviews was 106 min and the individual interviews, 50 min. We identified seven key themes linked to the three phases of the nurses’ involvement in the trial: pre-trial preparation; delivering the intervention and post-trial (Fig. 2).

We present our results in 3 sections to reflect the study aims. Verbatim quotes are identified as being from either PACE-Lift (PL) or PACE-UP (PU) trials, however these are not representative of individual nurses views, but have been chosen as the best representation of the groups’ view for each theme.

Aim 1: Perceived enablers and barriers to delivering the complex PA interventions.

### Pre trial

The pre-trial phase was characterised by the initial preparation and training. Both general training and support from the research team were experienced positively:

*“… it’s putting it into practice that is the difficult thing, so I think having the practical sessions, where we would role play, was really important… ”*(PU)

*“… you couldn’t actually do a programme like this unless that training was actually up front for anybody doing it.”*(PL)

However, there was a more mixed response to BCT-specific components of the training:

*“ I think, also, the [BCT] was very good, made you think and try and put it into practice more, that’s good, but a lot of jargon did come into it…the terminology and I don’t even remember the name of the words now, because I don’t understand them.”*(PL)

Ongoing support from the research team and peer support from other trial nurses was seen as “essential” and valued both before and during the intervention delivery.

*“…and it is good all to get together to see what’s happening in the other surgeries as well. If you’re struggling with something, you can see that other people are struggling.”*(PU)

To ensure the fidelity of the intervention and to provide feedback to the nurses, they each audio-recorded at least three of their patient consultations. Individual feedback was provided by the BCT trainer on aspects including the delivery, communication skills and appropriate use of BCTs. Once the initial feelings of self-consciousness were overcome, the nurses were unanimous in valuing this experience and felt that the feedback provided enabled them to improve their practice in future consultations.

*“I actually changed my practice from thereon, umm, so yes, it was … it was exceptionally helpful to listen to the reading.”*(PU)

However, several nurses identified that if there was a long delay between submitting a recorded consultation and receiving the feedback this detracted from the value of the exercise:

*“…it would have been much more valuable having that feedback earlier, umm, because, obviously, by the time I did get it, I’d seen a lot more clients umm … and perhaps I would have been able to have you know used those skills earlier with those other clients.”*(PU)

### Delivering the intervention

The nurses were scheduled to deliver the intervention in 4 (PACE-Lift) appointments of 20–30 min or 3 (PACE-UP) appointments of 10–20 min (PACE-Lift appointments were longer due to downloading of accelerometer data for discussion with participants). Length could be adjusted in response to patient needs and all agreed this approximate length was appropriate. The intervention incorporated BCTs, a PA diary, pedometer average daily steps (and accelerometer feedback on PA intensity in PACE-Lift). Individual PA plans based on increased walking and other existing PA were produced at each consultation. To ensure trial fidelity, the nurses were aware that they should adhere to the protocols, even if they had personal doubts about specific elements;

*“I was very aware that this is a trial and I have to do it, as it is stipulated”* (PL)

*“I did, of course, I had to use it, but I didn’t want to use it. I didn’t like using it but I had to use it.”* (PL)

However, our nurses perceived there to be ‘essential’ elements of the intervention and some which were ‘non-essential’. For example, rather than using the handbook verbatim, most had changed the wording or phrases to those with which they felt more comfortable;

*“Right from the beginning, the language in the book, I did not feel comfortable with. And so I picked out the essential bits from each session and made my own notes. And I followed those. Having … using all the essential bits, umm … but putting them in my own language.”*(PL)

*“..we had a set of handouts we could give to try and think of objectives or to try and look at negatives and positives, umm, I think I used them very, very minimal. The only time I ever did use them was for the very last session which sometimes I gave them so they could use … just reflect on it…”*(PU)

These comments reflected the views which had already been expressed at an earlier training session after nurses had worked with patients for 3 months post study initiation. It was therefore agreed with the trial team that the handouts would become ‘optional’ in order to maximise participant retention and engagement as well as nurse satisfaction with consultation delivery. This pragmatic amendment demonstrates the individual tailoring that the nurses were skilled at providing and which may have contributed to the successful outcomes of both trials.

Adherence to the protocol was also relaxed briefly due to a participant’s religious observance. This illustrates the flexibility and sensitivity required to tailor the intervention, while maintaining sufficiently uniform delivery:

*“… they couldn’t walk or increase on their walking at that time because they hadn’t eaten and then they weren’t feeling too good, and all that, so we did it a different way then, and what I did with them was we relaxed it and then I said to them, when Ramadan’s over, we made the appointment so that their actual trial went on a bit longer.”* (PU)

Seasonal variations, dark nights, poor weather and holiday periods were also raised as potential barriers to patients increasing their walking and the nurses had worked hard negotiating with participants to devise alternative strategies to encourage them over these periods;

*“If there is any trial, forget about the 2 months around Christmas …totally, because you can’t get appointments, and they don’t want to wear it …”*(PL)

*“…if the weather was bad, or it was cold, or there were obstacles that got in the way, they would umm … had a few people that umm decided to get their Wii Fit’s out and started doing more activity indoors as they were doing the Wii jog… so they would do things like, you know, doing things like activities indoors where they couldn’t always go outside”* (PU)

#### Intervention equipment

Full training was provided to the nurses on the use of pedometers and additional training was provided for the PACE-Lift practice nurses on how to use the accelerometers. The nurses were extremely positive about the pedometer, describing it as the *“motivation tool*” and a *“real incentive”*. The accelerometer was seen as an additional motivator in the PACE-Lift trial, as the nurses were able to use the graphing produced as a visual tool in each consultation. This allowed the nurses to demonstrate that despite walking the required number of steps some participants were not reaching the required intensity whilst walking;

*“… the equipment was excellent, the pedometers, the accelerometers, excellent, excellent, excellent. Because whilst you were looking for the time of walking, I turned it round into the quality of walking*.”(PL)

*“Pedometer could be on prescription as far as I’m concerned… I think it’s something that we should be using in GP practices… I think it actually shocks people into realising how inactive they are…then for the people that are active, I think it would increase their activity.”*(PL)

However, despite the accelerometer being highly rated by the nurses, it provided some technical challenges. They felt the computer system for downloading data was too complex and time-consuming within the time-limited consultation.

*“We had very good training for the computer programme but I struggled with it … I would have liked to have had an easier computer programme and spent more time looking at my patient and talking to my patient.”* (PL)

The equipment did not always record accurately due to individual participant characteristics:

*“I found that I had a particular lady who had umm … was quite disabled and walked in a … had a very unusual gait. The pedometer just didn’t work at all.”*(PL)

*“The pedometer didn’t always work….the obese patients and no waist - it didn’t work.”*(PL)

Some participants had to be actively encouraged to continue wearing the accelerometer as they reported problems with the belt. Choice of clothing when wearing a pedometer was found to be more of an issue for women:

*“They didn’t like the belt. None of them liked the belt.”*(PU)

*“Dresses were a problem weren’t they? Yes. The items of clothing that they were wearing, it was much easier for them to have trousers on…”*(PU)

#### Materials

A handbook, ‘Improving Health: Changing Behaviour’, (PACE-Lift and PACE-UP patient handbooks) was used by the nurses during consultations and a copy was provided to each participant at their first appointment for them to retain as part of the intervention. This was provided in A4 size in PACE-Lift, but feedback from the nurses indicated that the size was too cumbersome for participants. It was therefore reduced to A5 for PACE-UP (with A4 copies available if preferred).

There was mixed feedback on the materials used in the trials, which to some extent seemed to depend both on the nurse’s own perceptions and how they perceived their participant population:

*“…the only other thing I’d say about the diary is that the people that really liked filling it in found it a really good motivator. When they came to the last appointment, they wanted another one.”*(PU)

*“Well I always started off very enthusiastically about the book, and said this is your book, you know, and we’re going to go through it, and let me know what you think when you come back, you know, what was good and you fill it out yourself. I made it their book. Umm … and I had I would say three quarters of them come back and said, “I don’t need this.”*(PL)

#### Participant engagement

Almost all of the nurses felt it was a ‘privilege’ to have so much time to devote to participants and reported great satisfaction from witnessing a move towards healthier living:

*“… the people that have decided that they want to make their changes to their lives, and then having them come back, at their second, third appointments, and being so positive about everything, has been amazing and some of them have brought tears to my eyes…”*(PL)

*“I mean I still see patients regularly, that had been on the trial, and they still say, “Oh yes, I’m still walking”, you know, so you know, it’s good, yes, no - really good.”*(PU)

The nurses were not involved with the selection and recruitment of the participants; this was undertaken by a research assistant, who arranged the initial nurse appointment. Some nurses expressed doubts about the selection process for participants with many feeling that they were ‘too active’ for the trial or had agreed to take part for the ‘wrong’ reasons. Some nurses also felt that perhaps their regular ‘patients’ had volunteered to participate in order to ‘help them out’:

*“And then there were people that already had high activity levels that were kind of wondering, well how is this actually going to benefit me?”*(PU)

*“…I’d be interested to know, sorry, to know actually what happens after the year, because I completely agree in that a lot of them thought they were helping us.”*(PU)

#### Spousal couples

Over both trials, 105 spousal couples were recruited with 60 couples receiving the nurse intervention. Working with couples presented a unique challenge for some of the nurses as, in normal practice, it is unusual to be working with two patients simultaneously. Sometimes the dynamic worked well and other times the problems encountered were a significant barrier:

*“Most couples, they enjoy doing it together, because they’d go … they could go out walking together and, even if it was through the winter, at least if they were both going, they had each other.. they used to encourage each other. So if one didn’t want to go the other one would encourage them and they’d make sure they went.”*(PU)

*“I’m not actually overly sure how couples worked. I don’t know if I had … I don’t know if it caused more issues sometimes, in the fact with the pedometers, because they got so focused sometimes on the fact that their pedometers didn’t match up.”*(PU)

A summary of the enablers and barriers to delivering the intervention and recommendations by the research team to guide future development of the intervention and trial conduct are shown in Table 2

**Table 2:** Summary of the enablers and barriers to delivering the PA Intervention; recommendations for future studies

	Enablers identified by nurses	Barriers identified by nurses	Recommendations from the research team:
Pre – trial			
Training	• Comprehensive initial training day & follow up • Training delivered by ‘credible professionals’ • Clear instructions on what has to be carried out at each consultation	• Last minute cancellations by nurses due to work commitments	• Aim to recruit practices and nurses who are interested/committed to research (via PCRN’s) • Pre- trial preparation and training on all aspects of the trial by appropriately trained professionals is essential • Ensure a mix of both theory based and group role play sessions to allow nurses to rehearse difficult scenarios and to allow for reflection & discussion in a supportive environment • Importance of training and ongoing support should not be underestimated and care taken not to neglect the more ‘mundane’ elements of training (e.g. use of the computer) • Consider consulting potential trial nurses at protocol conception stage to gather their opinions on the intervention, recruitment of appropriate participants etc.
Specific training in behaviour change techniques	• Clear instruction by experienced trainer • Role play scenarios • Protocol guidance clear	• BCT terminology/ jargon sometimes not clear • Some role play scenarios too early as nurses had not seen participants yet	• Make sure any new terms and BCT jargon are clearly explained to the nurses and understanding is checked. • Role play, especially difficult scenarios is essential, However, time it appropriately in training
Support (from the research team and other practice nurses)	• Ongoing support • Accessible team members • Nurse group supervision • Communication		• Establish a good communication and support network/system, not only with the research team but also between the nurses in the trial but. • Communicate effectively with practices to enable nurses to attend training sessions etc. • Before a practice agrees to being involved, ensure protected time is negotiated for group/individual supervision
Delivering the intervention			
Timing between visits and length of consultations	• Length of appointments and timing between appointments just right	• Annual holidays and Statutory holidays delayed intervention timings (no appts available at surgery near Christmas)	• Consider whether the trial will take place over holiday periods. If so, have alternative strategies.
Seasonal variations & weather conditions	• Summer months & sunny weather	• Winter (due to darker evenings, snow and rain!)	• Consider timing of study • Use as a potential relapse/barrier & try to work with participants to find a solution to maintaining walking in poor conditions.
Feedback on performance during the intervention	• Being audio recorded and provided with constructive verbal and written feedback	• Felt self-conscious being recorded • Feedback not always timely	• Audio recordings of consultations a good way of ensuring quality and consistency across consultations • Ensure feedback is timely so that any changes can be made to consultations quickly • Ensure feedback is given both verbally and in writing
Following the trial protocol	• Clear protocols and guidance to follow at each consultation • Ability within protocol to individualise activity plans	• Religious observance (e.g. Ramadan) • Wording on some of the handouts patronising to some participants and nurses	• Consider the population area that you are recruiting from in terms of ethnicity and socio-economic groups • Allow some flexibility within the protocol to personalise the consultation to the participant without compromising fidelity • Consider the possibility that you may have to ‘relax’ the protocol in order to retain participants • Ensure nurses are aware of which elements of the protocol are essential for fidelity and which can be adapted – empower nurses to make ‘patient-centred’ changes where appropriate, to maximise trial retention and success
Use of equipment	• Pedometer • Accelerometer	• Pedometer not always accurate with participants who are obese, have unusual gaits or disabilities • Not able to wear pedometer easily with a dress • Differences in readings (especially couples) • Computer programme to extract accelerometer data too complicated and time consuming within consultation • Accelerometer belt uncomfortable to wear	• Be aware that certain participant characteristics may affect intervention • Consider admin staff time to support nurses to ensure quality of consultation • Explain that different people will record different step counts on the same walk, due to differences in stride length etc. • Consider use of equipment not worn on a belt, such as accelerometers within smartphones etc.
Use of materials -Handbook, handouts and diaries	• Patients enthusiastic about step count diary • Freedom to individualise goal setting targets within consultation	• Terminology and content of some of the handouts & handbook off-putting • Handouts too general	• Ensure that all materials are piloted with appropriate groups before trial starts • Allow flexibility on use of materials if individuals do not find them helpful
Participant engagement	• Motivated participants	• Some participants considered too active • Patients not committed to long term change • Patients ‘complying’ to ‘help the nurses out’	• If possible, involve nurses in participant selection & recruitment and if not possible, then ensure nurses are fully aware of inclusion/exclusion criteria so they are reassured that the correct patients are recruited • Consider appropriate exclusions, particularly for PA trials
Spousal couples	Often couples motivated each other to walk more	• Difficulties dealing with couples requires additional training	• If considering an intervention aimed at couples, the nurses will require more training & support to build confidence as this is a novel way of working and has complexities not dealt with simply by giving more time within the consultation

### Post-trial

#### Changed practice

Regardless of previous BCT or experience of research trials, all the nurses spoke about personal development and enhancement of their knowledge and skills. These skills were identified as being extremely useful techniques to use across a wide range of routine lifestyle consultations, not just PA, but also smoking cessation, weight loss management and in the prevention of chronic diseases:

*“…I think that really helped me to listen and to ask the open questions, and then wait for them to talk back to me, rather than me going … and to not drop into nurse mode…”*(PL)

*“…the golden silence, that’s what I take away from PACE, I have learnt to shut up and not spoon-feed, believe it or not”*(PL)

Most felt that it had increased their confidence and had ‘transformed’ the way they practised in routine consultations, to the benefit of their patients

*“…now I’m pretty damned slick and I’m enthusiastic and I know exactly what I’m talking about and what I don’t need to be talking about, which is where the BCT comes in…”*(PL)

*“Definitely use the techniques in other consultations. I think the confidence ruler has become very much part of my consulting, actually, now. …”*(PU)

There was overall agreement that their routine consultations had become more patient-centred:

*“…the good thing about [BCT] that I’ve learnt is actually about leaving it to them, and that’s been the biggest learning curve for me, and it’s still not easy, but like, things like the pauses and you know … waiting for them to come up with solutions, has probably been the biggest impact on my practice”* (PU)

*“…sometimes they come up with the solution that, you know, you haven’t even thought of, and that’s … that’s what it’s about isn’t it really, you know, they come up with their own ideas.”*(PU)

There was unanimous agreement that their involvement in the trial had enabled them to spend valuable time with participants;

*“It has been a luxury…and we’ve enjoyed it…. a fantastic luxury…being paid to do what we love doing. And to actually have time to do it … to communicate with patients, to listen to them, and actually figure out what their agenda is, and help them achieve their goals. You can’t do that in a normal consultation.”*(PL)

*“…you know we don’t have any protected time for health promotion. Our role is clinicians…the health promotion is the add-on… it’s giving us the time to do it because we don’t have the time.”*(PL)

However, although acknowledging that the intervention would be beneficial to their patients, they observed that within the time constraints of routine practice they would not be able to undertake the full intervention as it stood within a routine nurse consultation:

*“We have 10 min to see a patient who comes to see us for whatever, to do everything, and, Oh, by the way, how much are you eating, drinking, walking, smoking?”*(PL)

It was suggested that the current trial material could be adapted to incorporate the pedometer and printed materials for use within consultations and perhaps be available on prescription:

*“…if you had, in your drawer, you had like a set … a package, programme, you could do, and if through the NHS Health Check you identified someone who was suitable, you could then discuss it with them and say, “Would this be something you’d be wanting to look at?” and go from there.”*(PU)

The possibility of training a health advisor or health facilitator to deliver the intervention as it stood instead of a nurse was met with some skepticism. The nurses reflected on their professional training, the tacit knowledge, skills and experience which had enabled them to make autonomous decisions in order to individually tailor and to adjust the interventions for their patients.

*“I personally think that intervention, with the practice nurse, is invaluable. It … it was … we know what we’re talking about.”*(PL)

*“That’s the thing about practice nurses. We’re used to working on our own, with our own patients.”*(PL)

## Discussion

### Principal findings

There was unanimity that participating in the trials was a positive experience and that the nurses saw themselves as an essential element of the interventions. Other important enablers included: the comprehensive pre-trial preparation; the research team support and feedback from BCT trainers; the peer-support from the other trial nurses; and the motivation provided by the equipment. The BCT training was particularly valued for personal development and had enabled the nurses to integrate the BCTs confidently & effectively into their routine patient consultations.

Important barriers to successful intervention delivery included difficulties adapting trial materials to particular patient groups; uncertainty about which (if any) elements of the protocol were ‘optional’; equipment and technology problems; managing seasonal variations in weather; holidays; and dealing with couples. The nurses felt that their expertise had enabled them to deliver the intervention effectively, but time constraints would make delivery difficult within routine consultations.

### Comparisons with other studies

Consistent with the findings of Boase et al. [37] the practice nurses viewed themselves as an essential element of the intervention, more specifically, the ‘key to the delivery of the trial’. Our findings concur with others, that participating had changed their approach within routine patient consultations by becoming more patient-centred, allowing patients to take more responsibility for changing their health behaviours rather than ‘dropping into nurse mode’ and providing all the answers [24, 38]. We also agree that in delivering an intervention, nurses should be appropriately trained and supported before and during delivery [24, 39] and that additional time be taken to explain the specific language used within the BCT training [37].

The nurses enjoyed having increased time with the participants and felt gratified that their enhanced behaviour change skills and knowledge was transferable to routine consultations [24, 37, 39]. However, they doubted that they would be able to undertake the complex intervention ‘as it stands’ within a routine nurse consultation in a GP setting, due to time constraints [14, 37, 40].

Our findings support the use of pedometers with a package of support and monitoring to motivate patients to increase PA [14, 24, 41]. However a degree of flexibility in the design and implementation of the intervention is needed to ensure that it can be adapted to both local circumstances and patients’ cultural needs [23, 25, 27].

There were some relationship difficulties working with patients who became participants and the nurses were also unsure whether appropriate patients had been recruited [37]. They had not been involved in the recruitment process and our findings concur with others [39] that staff who deliver an intervention should be involved from the beginning, asked for their opinions, and constantly updated on trial progress to maintain their enthusiasm.

Whilst observational work suggests that spousal couples are very important in initiating and maintaining important health behaviour changes [42], there are very few behavioural intervention studies which recruited spousal or co-habiting couples [43, 44]. To our knowledge this is the first time the experiences of delivering a behaviour change intervention to couples within the same consultation in a PA intervention study has been reported.

### Strengths and weaknesses

#### Strengths

The findings reported are from practice nurses who delivered around 1400 complex PA health behaviour change consultations to over 500 participants. The two trials covered different geographical areas and included both men and women, with an age range from 45 to 75 years and from a range of different socio-economic and ethnic backgrounds. The practice nurses were also diverse in terms of age and experience. The broad similarity of the findings from both trials adds credibility to our findings and maximises their transferability to other interventions and settings.

Two of the researchers were involved in both focus groups and one undertook the individual interviews, providing some consistency. The group interviews (and the analyses) were led by an experienced qualitative researcher (CV) and involving the wider research team in reaching a consensus supported the credibility of our interpretations and enhanced the dependability of the findings. The nurses had supported each other throughout both trials and were comfortable in each other’s company during the interviews. The researchers who conducted the interviews were not known to them and they were therefore able to freely express their honest views and opinions without fear of offending the people who had supported them during the trial, and without assumptions about views and experiences already expressed in previous meetings with trial personnel. The draft manuscript was circulated to the nurses for review of accuracy prior to submission and no amendments were suggested, increasing authenticity of the data.

#### Weaknesses

Although every effort was made to engage all 12 nurses, one nurse was unable to participate and may have expressed different views, potentially affecting the findings. However this nurse joined late in the trial and only saw a very small number of participants. It could also be seen as a weakness that around half of the nurses had previously participated in research and/or BCT training. These more experienced practitioners may have been more confident, able to make independent decisions and be flexible with the protocol as opposed to nurses without this previous experience.

Group interview methodology presents a potential risk of a ‘dominant voice’ unduly influencing the overall findings. However, our groups were facilitated by researchers with extensive experience and we found substantial agreement between the themes emerging from both groups and with the individual interviews, supporting the validity of our findings*.*

### Recommendations for practice and research

Complex intervention studies in general practice delivered by practice nurses are becoming more commonplace. However, there is a need to gather insights from those who deliver the interventions to provide a more complete understanding of both the process and the results.

As summarised in table 2, our study suggests that, at the pre-trial stage, investigators should aim to recruit practices and nurses who are interested in research; involve the nurses at the earliest stages of trial planning; ensure training is comprehensive, responsive and delivered by skilled professionals; and establish a good communication and support system for the nurses, which should be maintained throughout the trial.

Our study identified many potential recommendations regarding intervention delivery, including: considering carefully the seasonal timing of the intervention; implementing strategies to deal with barriers such as inclement weather, holiday periods and fasting; use of consultation audio-recording with timely feedback to ensure quality and consistency; allowing appropriate flexibility, without compromising trial fidelity, in the protocol to accommodate participant preferences and lifestyle and communicating this effectively to trial nurses; ensuring that all materials are piloted widely with appropriate groups before the trial starts; and acknowledging that if an intervention is to include couples, the nurses will require more training and support to manage this confidently. Many of these recommendations hold not just for interventions delivered in research settings, but also to those delivered in routine care and may be of relevance to the NHS Diabetes Prevention Programme which will include interventions to target physical activity and is due to be rolled out through primary care in England [45].

The training of the practices nurses in BCTs was an important part of both trials. Latest recommendations advocate BCT training for lifestyle modification for all health and social care staff with roles having the potential to influence and encourage behaviour change [46]. Practitioner confidence and knowledge has been found to affect the ability to discuss and/or prescribe PA, with the main reason cited for low confidence being a lack of specific training [5]. This training should be delivered by ‘credible experts’ [47] and include key competency assessments of brief interventions and motivational interviewing techniques for lifestyle modification [3].

Due to a combination of their experience, tacit knowledge and skills acquired from the BCT training, the nurses in this trial were likely to be practising at level 4 of this competency framework, using “specialist/advanced or lifestyle and behaviour specific behaviour change approaches to support individuals…” [48]. They used these skills to decide which aspects were core to the trial and which were less essential and were able to adapt and tailor the consultations. In contrast, health care assistants/facilitators/practitioners may achieve lower competency training at levels 1 or 2 and would provide ‘opportunistic brief advice or undertake a brief intervention’, which requires a ‘superficial understanding of principles or theories’ as opposed to level 4, which demands the ‘application of factual knowledge in a manner that takes account of widely understood principles or theories and implications within the field of practice’. This supports the use of practice nurses in such trials and the mainstreaming of such interventions.

## Conclusions

Complex PA interventions have multiple elements which can present as both enablers and barriers and therefore we need to ascertain which elements within the complex intervention might be adapted for successful use within routine consultations. We have demonstrated that practice nurses are positive about delivering PA interventions with the right materials and support. They already have professional experience and skills, which are enhanced by training from credible sources, but they require clearer guidance on the extent to which they can modify the intervention to meet individual needs, without jeopardizing fidelity. The transferable skills gained from the training and participation in the trials had ‘transformed’ the way the nurses practised within routine consultations to the benefit of all their patients and although they felt that the intervention could not be delivered in its current form within a routine 10 min consultation, they recognised that certain aspects, such as the handouts, could be used opportunistically in future consultations.

These findings further develop the evidence base on the effectiveness of healthcare and public health interventions. The lessons learnt from this qualitative evaluation can be used to guide and inform researchers or policymakers in order to tailor and enhance the development and delivery of future health behaviour change interventions or programmes delivered by practice nurses in primary care, and the conduct of related trials.

## Appendix 1

### Nurse group interview schedule

#### Introductions

*Introduce ourselves.* Explain one will lead while the others take notes, just in case the recording fails etc.

*The aim of this session* is to find out what it was like to be involved in this study and to help patients to increase their physical activity using this particular method and this schedule of visits. It’s important that you tell us what it was like, warts and all – so that we can let the team know what went well and what could have been better both for you and for your study patients. It’s also important that we have all of your views – so if you disagree with what someone has said, then make sure we hear your perspective too. Hopefully, it will be a discussion between all of you, with us just throwing in a few questions to keep things going. OK?

*Anonymity and confidentiality.* Now say we won’t use their names if we extract something they said for a paper – also, nobody but us will know who said what, and it won’t be passed on to other members of the team.

*Tell them we’re turning on the recorders.*

*Ask the nurses to introduce themselves for the recording.*

*----------------------------------------------------------------------------------------------------------------------------------------------------*

**The Interview schedule***(prompts in italics)*

1. **What stood out most for you from the actual training sessions?**
  - *What specific parts of the training do you recall as being particularly useful? (Challenge if they say ‘all of it!’ – must be precise)*
  - *Was anything less useful or could be improved?*
  - *Would you have liked anything more in the way of training or materials?*
  - *What do you feel about the number of training sessions? (too many / not enough / about right?)*
  - *What did you feel about the balance of the training sessions between communication / behaviour change techniques and practical trial aspects (physical activity guidelines /using pedometers / handbooks / reporting adverse events etc.).*
2. **What was it like using the handbook/diary?** (remind the nurses about the handbook/diary by showing it to them –have one copy to look at together, or else it will turn into individual silent reading sessions)
  - *How did you find using it?*
  - *How did the patients find it? What were the best bits? Which bits caused most difficulty? How did you get round this?*
  - *How could the handbook be improved?*
3. **What about using the pedometer?**
  - *How easy was it to explain to people how to use it?*
  - *How common were difficulties with the pedometer? What kind of difficulties did people have?*
  - *Were most people happy to wear the pedometer whilst coming to see you & keep a step-count record?*
  - *Were targets that we had suggested realistic for most people? Did a lot of people change their targets? If people changed them did they tend to set higher or lower targets?*
4. **How acceptable did patients find the intervention?**
  - *What were the characteristics of someone who really ‘went for it’?*
  - *Were any patients more responsive to the PA intervention than others*
  - *What about the characteristics of someone who really didn’t get on with it?*
  - *Did anyone say anything to you that hinted at why they didn’t like it?*
  - *Did you have experience of working with couples in the trial? How did you find this? What were the positive aspects? And the negative?*
5. **How about the trial protocol - the schedule for seeing patients (3 visits a month apart, first visit approximately 30 min, others approximately 20 min).**
  - *Was it possible to do what was required of you in the time prescribed?*
  - *Were there enough sessions/too many?*
  - *Did most patients actually get the 3 visits at the right time? How did it vary between patients?*
  - *Did you have problems with non-attenders? How did you manage this?*
  - *If things went wrong, how easy was it for you to get help / support from the study team?*
  - *How did you feel about having some of your sessions recorded? Was recording them or receiving feedback on the sessions helpful?*
6. **Some of you are also involved in NHS health checks at your practices, do you see this intervention as something that could be useful for those identified in health checks as needing to increase their physical activity levels?**
  *If yes, how could this work? If no, why not?*
7. **From the nurse perspective, if we were to do the trial again with different practices, or try to put the intervention into your routine practice…**
  - **What would be the main things to keep?**
  - **The main things to change?**
8. **And from the patients’ perspective, as far as you can tell**….
  - **What would be the main things to keep?**
  - **The main things to change?**
9. **Did your physical activity consulting change as a result of the training or from being involved in the trial?**
  - *If so, how? If not, why not?*
10. **Have any other aspects of your work changed?**
11. **Anything else that you think we have missed / that you want to tell us?**
